# Automated quality indicators for echocardiographic recordings and measurements: consequences for left ventricular global longitudinal strain

**DOI:** 10.1093/ehjimp/qyag124

**Published:** 2026-07-20

**Authors:** Even Olav Jakobsen, Andreas Østvik, David Pasdeloup, Erik Smistad, John Nyberg, Stian Stølen, Lasse Lovstakken, Espen Holte, Bjørnar Grenne, Havard Dalen

**Affiliations:** Department of Circulation and Medical Imaging, Norwegian University of Science and Technology (NTNU), Box 8905, Trondheim 7491, Norway; Department of Cardiology and Cardiothoracic Surgery, St. Olavs Hospital, Trondheim University Hospital, Box 3250 Torgarden, Trondheim 7006, Norway; Department of Circulation and Medical Imaging, Norwegian University of Science and Technology (NTNU), Box 8905, Trondheim 7491, Norway; Medical Image Analysis, Health Research, SINTEF Digital, Trondheim, Norway; Department of Circulation and Medical Imaging, Norwegian University of Science and Technology (NTNU), Box 8905, Trondheim 7491, Norway; Department of Circulation and Medical Imaging, Norwegian University of Science and Technology (NTNU), Box 8905, Trondheim 7491, Norway; Medical Image Analysis, Health Research, SINTEF Digital, Trondheim, Norway; Department of Circulation and Medical Imaging, Norwegian University of Science and Technology (NTNU), Box 8905, Trondheim 7491, Norway; Department of Cardiology and Cardiothoracic Surgery, St. Olavs Hospital, Trondheim University Hospital, Box 3250 Torgarden, Trondheim 7006, Norway; Department of Circulation and Medical Imaging, Norwegian University of Science and Technology (NTNU), Box 8905, Trondheim 7491, Norway; Department of Circulation and Medical Imaging, Norwegian University of Science and Technology (NTNU), Box 8905, Trondheim 7491, Norway; Department of Cardiology and Cardiothoracic Surgery, St. Olavs Hospital, Trondheim University Hospital, Box 3250 Torgarden, Trondheim 7006, Norway; Department of Circulation and Medical Imaging, Norwegian University of Science and Technology (NTNU), Box 8905, Trondheim 7491, Norway; Department of Cardiology and Cardiothoracic Surgery, St. Olavs Hospital, Trondheim University Hospital, Box 3250 Torgarden, Trondheim 7006, Norway; Department of Circulation and Medical Imaging, Norwegian University of Science and Technology (NTNU), Box 8905, Trondheim 7491, Norway; Department of Cardiology and Cardiothoracic Surgery, St. Olavs Hospital, Trondheim University Hospital, Box 3250 Torgarden, Trondheim 7006, Norway; Department of Medicine, Levanger Hospital, Nord-Trøndelag Hospital Trust, Kirkegata 2, Levanger 7600, Norway

**Keywords:** GLS, left ventricle, deformation imaging, artificial intelligence, cardiology

## Abstract

**Aims:**

Global longitudinal strain (GLS) is a sensitive measure of left ventricular (LV) function, though hampered by significant variability. Data on how echocardiographic recordings and measurements influence GLS and its variability are scarce. We aimed to study the consequences of variability in echocardiographic recordings and measurement procedures for GLS in a normal population.

**Methods and results:**

Healthy participants (*n* = 1412) from the Trøndelag Health Study were examined by echocardiography according to current recommendations. Four experienced operators read and re-read all GLS recordings (2824 measurement procedures). Characteristics of echocardiographic recordings and measurement procedures were extracted by deep learning (DL) and software metadata.

LV apical endocardium was positioned at a mean distance of 26 mm from the top of the sector and 6 mm to the left of the central axis. Mean LV foreshortening ranged 1.2–2.1 mm in apical views and the recordings were well standardized with the preferred cut-planes for tilt and rotation. GLS (in percentage points) was lower with reduced image quality (0.8), longer LVs (0.2 per cm), wider regions of interest (0.8 per mm) during the measurement procedure, and longer distance (0.07 per mm) from the transducer to the LV apex, respectively. Moreover, GLS was 0.7 percentage points higher per mm apical foreshortening, and also higher when a more refined ROI initialization was used.

**Conclusion:**

Using novel DL methodology and available metadata, we found that indicators of image standardization and measurement procedures influenced GLS. Implementing such quality indicators during recording and measurement procedures may improve the efficacy of echocardiography and ease the interpretation of differences between studies.

## Graphical Abstract

**Graphical Abstract:**
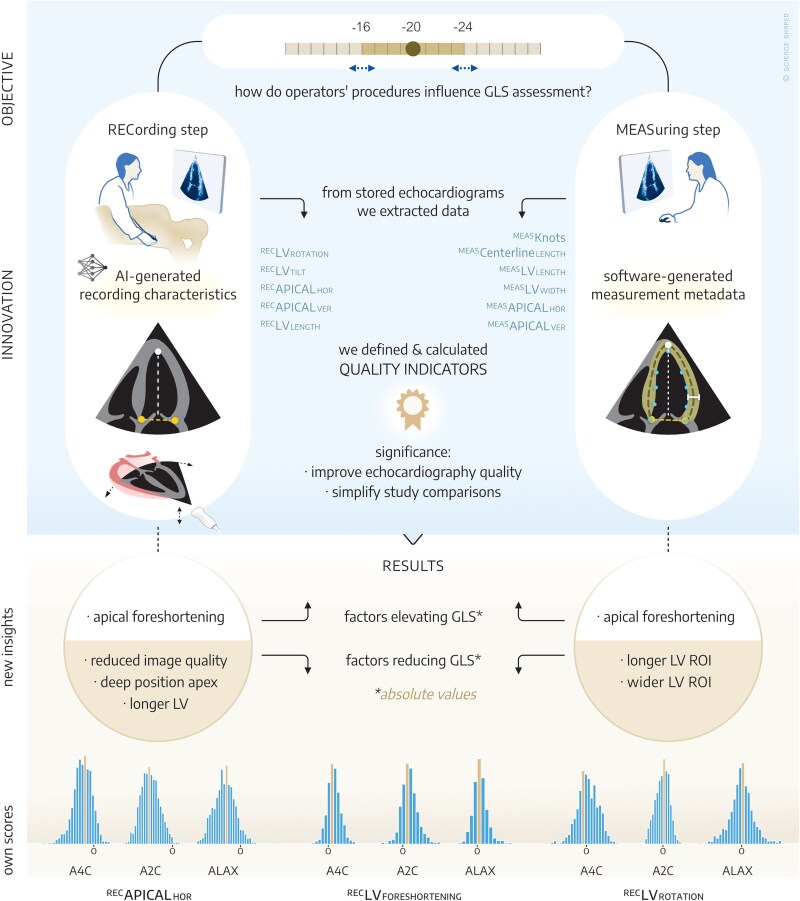

## Introduction

In clinical echocardiography, quantification of left ventricular (LV) size and function is essential. One of the guideline-recommended measures of LV function is myocardial deformation (strain), which offers valuable diagnostic and prognostic insights compared to LV ejection fraction.^1–3^ The most used strain measure is peak systolic global longitudinal strain (GLS). GLS has better test–retest reliability compared to LV ejection fraction and is considered the most sensitive and reliable echocardiographic measure to identify subtle changes in LV function.^4,5^ To accurately identify myocardial dysfunction, it is vital to know the normal ranges for a specific method in a healthy population. Reducing variation may improve the differentiation between normality and pathology. In large populational studies, lower GLS (in absolute values) was associated with higher age, male sex, and larger measures of body size.^6–8^ Other factors that may increase the variability include vendor-specific differences between ultrasound scanners, variations in echocardiographic acquisitions (recordings), between- and within-operator differences in the data analyses (measurement procedures), country- and hospital-specific biases and a limited number of participants per study site.^1,9^ To reduce variability, it is important to standardize the whole workflow, including recordings and the image analysis (e.g. strain measurements).^9^ With respect to image recordings, it has been shown that if the apical recordings are foreshortened, the strain measurement will be overestimated.^10^ Moreover, view-specific image quality and alignment with the optimal cut-plane in the standardized apical views is difficult to measure.^11,12^ GLS analysis may also introduce variation. First, at initialization of the region of interest (ROI), with adjustment of the position of apical and basal ROI points. Secondly, by adjustment of the ROI width to cover and subsequently track the myocardial deformation. As no user support is currently available to aid operators in optimizing echocardiographic recordings, it is left to the operator to decide whether the image recordings and measurement procedures are adequately standardized. To the best of our knowledge, the importance of quality indicators from image recordings and the process of performing GLS measurements have not been published for high-end echocardiography.

Fully automated deep learning (DL) tools to aid the operator in obtaining optimized echocardiographic recordings during live scanning have been developed.^13,14^ Importantly, these tools enable retrospective analyses in stored echocardiograms, allowing for fully automated quantification of apical positioning, LV foreshortening, and deviation in rotation and tilt from the preferred cut-plane of three standard apical views. Additionally, analysis-specific metadata from the GLS analysis software can provide detailed information on operator-specific strain measurement procedures.

Our aim was to study the consequences of differences in image recordings and the measurement procedures for GLS in a healthy population by utilizing automated image analysis by DL and analysis-specific software metadata. Furthermore, we aimed to explore the influence of these differences on reference ranges for GLS and on the measurement variability between operators.

## Methods

### Study population

**Figure 1:**
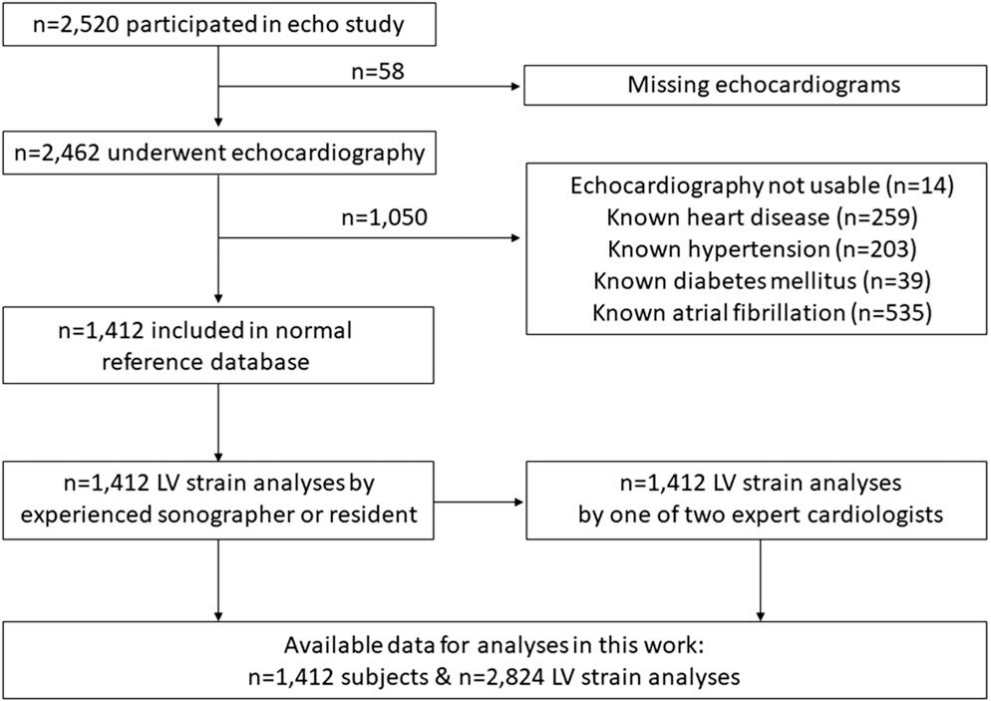
Study flow chart. Abbreviations: LV, Left ventricle; *n*, number.

Comprehensive details of the study population are previously published.^8,15,16^ The population was recruited from the fourth wave of the Trøndelag Health Study (HUNT4), Norway, performed 2017–19.^15^ A total of 56 044 (54% of invited) individuals participated in the HUNT4 baseline study and a subsample of 5763 participants in the baseline population was invited to the HUNT4fitness and echocardiography study. Inclusion criteria were participation in the HUNT4 baseline study and either HUNT3 Fitness or Echocardiography study (2006–2008), having a validated atrial fibrillation diagnosis from HUNT3, or self-reporting atrial fibrillation at the HUNT4 baseline examination. In total, 3174 responded to the invitation, whereof 2462 persons participated in the HUNT4Echo Study. In the present study, 1050 subjects were excluded due to no readable echocardiogram, known heart disease, hypertension, diabetes, or atrial fibrillation (*Figure 1*) and 1412 (55.8% women) were included in the analyses. The basic characteristics of the study population were recently published.^8,16^ Details are summarized in *Table 1* for the completeness of the presentation.

**Table 1:** Basic characteristics of the study population

	*n*	Overall (*n* = 1412)	Female (*n* = 788)	Male (*n* = 674)	*P* (sex)
Age, y	1412	57.5 (12.4)	57.2 (12.4)	57.8 (12.4)	0.040
Height, cm	1411	172 (9)	166 (6)	179 (6)	<0.001
Weight, kg	1411	76.4 (13.7)	69.8 (11.6)	84.7 (11.5)	<0.001
Body mass index, kg/m^2^	1411	25.8 (3.6)	25.3 (3.9)	26.3 (3.2)	<0.001
Body surface area, m^2^	1411	1.89 (0.20)	1.77 (0.14)	2.04 (0.15)	<0.001
Systolic BP, mm Hg	1411	128 (18)	127 (18)	131 (17)	<0.001
Diastolic BP, mm Hg	1411	75 (10)	72 (9)	78 (10)	<0.001
Heart rate, beats/min	1408	66 (11)	68 (11)	64 (11)	<0.001
Current smoker, *n* (%)	1404	37 (3%)	28 (4%)	9 (1%)	0.014
Never smoker, *n* (%)	1404	720 (51%)	389 (50%)	331 (53%)	0.145
*Echocardiographic indices*					
LVEDV, mL	1309	110 (31)	95 (22)	128 (30)	0.001
LVEF, %	1309	60 (5)	60.4 (4.8)	59.6 (5.1)	0.006
GLS, 18 segments	1278	19.7 (2.1)	20.1 (2.1)	19.2 (2.0)	0.001

Data are presented as mean (standard deviation) if not stated elsewhere. Similar data are previously published.^8,16^ However, in the publication by Nyberg *et al*.^8^ 83 individuals with systolic blood pressure >160 mmHg at baseline examination were excluded. Abbreviations: BP, blood pressure; LVEDV, left ventricular end-diastolic volume; LVEF, left ventricular ejection fraction; GLS, global longitudinal strain; *n*, number.

### Echocardiography

Details of the echocardiographic recordings and the strain measurement procedures have recently been published^8^ but are summarized in *Table 2* and the Supplementary material for the completeness of the presentation. Two experienced sonographers performed all echocardiographic scanning using GE Vivid E95 scanners (GE HealthCare, Horten, Norway) during 2017–2018, whereof one (KS) performed 1068 (75.6%) and another (EOJ) performed 344 (24.4%), with no significant differences across sexes (*P* = 0.23). The strain measurements were done off-line using EchoPAC SWO (version 204; GE HealthCare).

**Table 2:** Level of expertise and distribution of analyses by operator

Operator	Level of expertise	Experience with strain (years)	Number of analyses
Resident (JN)	Intermediate (>500 recordings/readings). ASE II, except for TEE.	3	958 (30)
Sonographer (EOJ)	Experienced (>2000 recordings/readings). ASE III, except for TEE.	5	454 (30)
Expert (BG)	Expert (>10 000 exams, >5000 strain analyses). ASE III. EACVI certified (TTE).	>15	683 (60)
Expert (HD)	Expert (>10 000 exams, >5000 strain analyses). ASE III. EACVI certified (TTE + TEE).	>15	729 (60)

Operators (initials) with description of level of experience and analyses shown as numbers read by each operator in the normal population sample (reproducibility sample). Years refer to the years of experience with strain imaging. Abbreviations: ASE, American Society of Echocardiography level of experience; EACVI, European Association of CardioVascular Imaging; TEE, transesophageal echocardiography; TTE, transthoracic echocardiography. Modified from Nyberg *et al*.^8^ with permission under the terms of the Creative Commons CC-BY license.

### Evaluation of echocardiographic recordings and strain measurement procedure

We employed two DL-based image analysis tools that have been developed and validated by our group: (i) *foreshortening calculation software*, which uses DL-based segmentation to measure view-specific LV length, apical and basal positions, as well as LV foreshortening,^14,17^ and (ii) *rotation and tilt estimation software*, which assesses the alignment of the three standard apical views in relation to deviations from the optimal view-specific cut-plane.^13^ Information of the apical and basal position of the LV, the axis of the cut-plane of the LV, as well as the alignment of specific apical views (A4C, A2C, and ALAX) with respect to rotation and tilt were extracted by the DL-tool from all recordings.

### Recording-specific (REC) quality indicators

**Figure 2:**
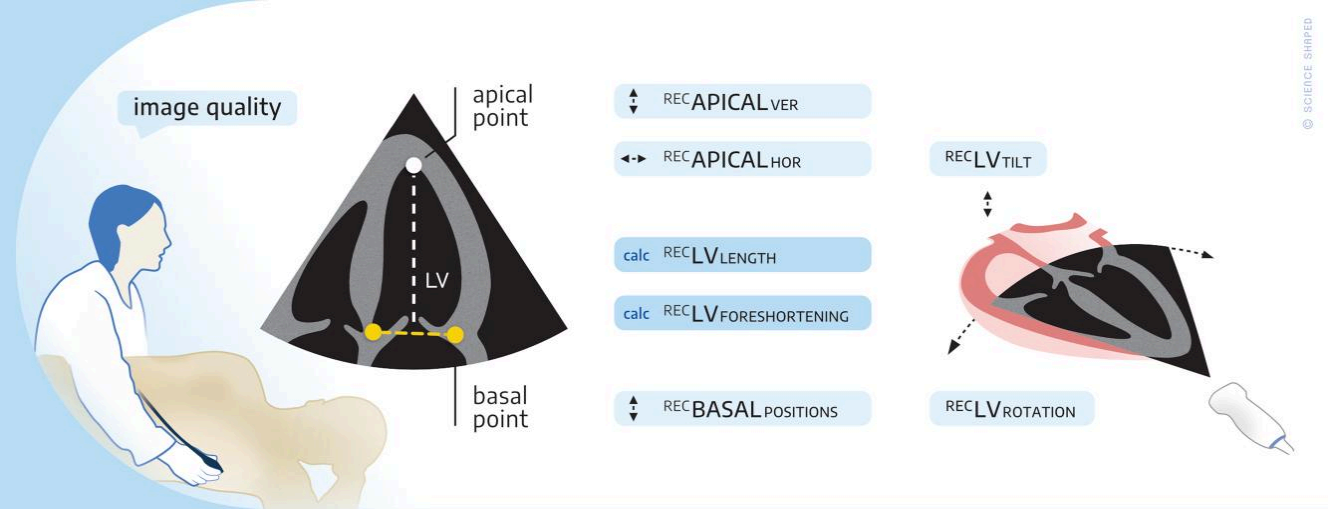
Details of image recording-specific data extracted by deep learning. Recording-specific data extracted from the images using deep learning software. The measurements were directly extracted from the recordings and the calculations were derived from the measurements. *Table 3* includes the specific explanations and definitions of the measurements and calculations used in this work.

*Figure 2* and *Table 3* show the details and definitions of the recording-specific data (REC) extracted by the DL software. The positions of the LV apical and basal points in the ultrasound image were quantified by the software. Extracted previews from all view- and cycle-specific outputs at ED and ES used for GLS measurements (*n* = 16 944) were overseen by two operators (EOJ and HD). Recordings where the segmentation had failed were excluded (see Supplementary data online, *Figure S1*). Apical foreshortening (^REC^LV_FORESHORTENING_) in each of the three apical views was calculated as the vertical movement of the apical endocardial point during systole. Positive values indicate downward movement, i.e. foreshortening, and vice versa. Similarly, the view-specific LV length (^REC^LV_LENGTH_) was calculated as the distance between the apical endocardial point and the centre of the calculated basal line between the two mitral annular points. The deviation in rotation and tilt between the actual two-dimensional cut-plane of the recording and the desired view-specific reference cut-plane (^REC^LV_ROTATION_ and ^REC^LV_TILT_, respectively) was defined and measured as described in *Table 3*. Importantly, metrics were extracted per A4C, A2C, and ALAX view, respectively, and averaged global values were calculated.

**Table 3:** Definitions and details of recording specific (REC) quality indicators

Abbreviation	Definition
Image quality	Judged by the two of the operators (JN or EOJ) as reduced (score = 2) if expected to influence LV strain analyses, otherwise scored as preserved (score = 1).
^REC^APICAL_VER_	Vertical distance from the transducer to the apical endocardial point in mm.
^REC^APICAL_HOR_	Horizontal deviation from mid of sector to the apical endocardial point in mm, where negative values indicate leftward deviation and positive values indicate rightward deviation.
^REC^LV_FORESHORTENING_	Apical systolic displacement in mm calculated from vertical movement of the apical endocardial position. Positive values indicate foreshortening (downward movement in sector), while upward movement in the sector during systole is provided as negative values.
^REC^LV_LENGTH_	LV length in cm measured from the apical endocardial point to mid of the basal centreline.
^REC^LV_ROTATION_	Rotational deviation from the optimal cut-plane. For each of the three apical views, a score of 0 indicates perfect alignment, while negative values indicate clockwise rotation of the transducer and positive values indicate counter-clockwise rotation of the transducer. The rotation between A4C and A2C or A2C and ALAX corresponds to a score of approximately 2. E.g., an A4C recording located in the middle between A4C and A2C will have a score of 1.
^REC^LV_TILT_	Deviation in tilt of the cut-plane from the optimal cut-plane. Positive values indicate deviation in the anterior direction for A4C, septal direction for A2C, and inferoseptal direction for ALAX, and vice versa. The values for this score are customized and standardized where the score relate to the probability and direction of correctly indicating misalignment from the optimal cut-plane. Thus, the score for tilt could not be easily aligned to specific cardiac structures.

Image quality was assessed by two operators as described, while ^REC^LV_ROTATION_ and ^REC^LV_TILT_ were extracted by the rotation and tilt software.^13^ All other variables were extracted using the Foreshortening software.^14^ Abbreviations: A2C, apical 2-chamber view; A4C, apical 4-chamber view; ALAX, apical long-axis view.

### Measurement procedure (MEAS) specific quality indicators

From the regions of interest (ROIs) placed and adjusted by the operator during the strain measurement procedure (MEAS), we extracted information, including the positions of the apical and basal points, the LV length, and the length and width of the ROIs.

**Figure 3:**
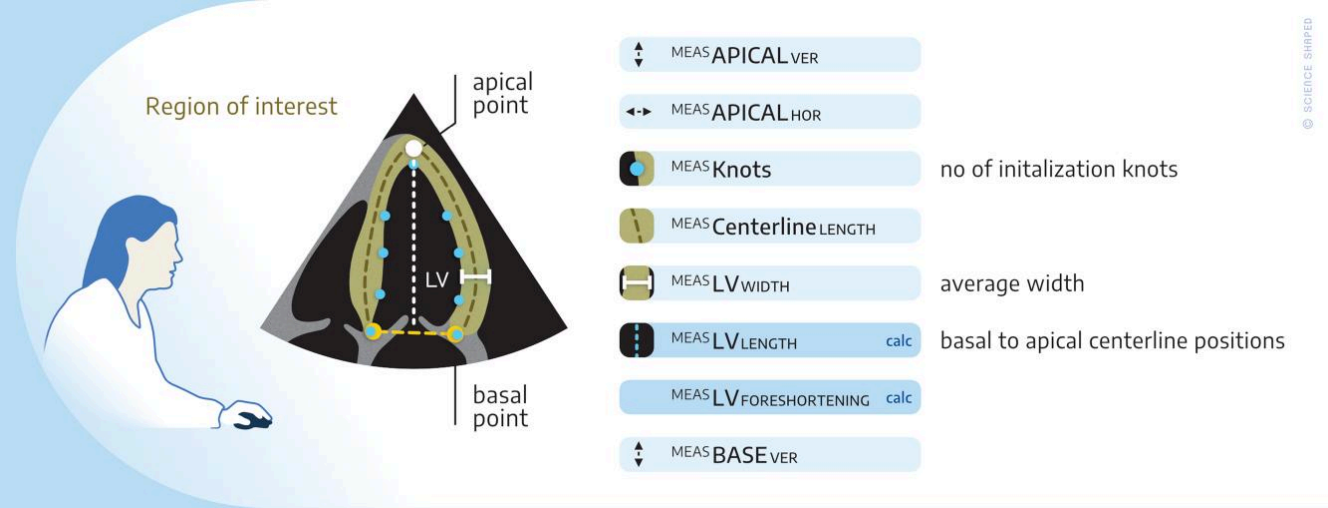
Details of measurement procedure-specific data extracted from the GLS analyses. Measurement procedure-specific data extracted from the strain analyses. Measurements were extracted directly from the GLS metadata, and the calculations were derived from the measurements. *Table 4* includes the specific explanations and definitions of the measurements and calculations used in this work.

*Figure 3* and *Table 4* provide definitions and details of the metadata used as quality indicators. These metadata were extracted from all GLS measurements procedures and included details of the number of knots or landmarks placed on the endocardial by the operator to initialize the ROI (^MEAS^Knots), as well as the mean width of the ROI from endocardium to epicardium (^MEAS^LV_WIDTH_) and the length of the LV ROI (^MEAS^LV_LENGTH_) as measured from the apical ROI centreline point to the mid of a calculated line between the mitral annular points. Additionally, we extracted data on the full length of the inverse U-shaped ROI centreline (^MEAS^Centreline_LENGTH_), along with the vertical and horizontal positions of the ROI centreline points at the apex (^MEAS^APICAL_VER_ and ^MEAS^APICAL_HOR_, respectively) and the two basal points. Using the movement of the ^MEAS^APICAL_VER_ through systole, we calculated the apical foreshortening of the ROI (^MEAS^LV_FORESHORTENING_), with downward motion denoting foreshortening and vice versa.

**Table 4:** Definitions and details of measurement procedure specific (MEAS) quality indicators

Abbreviation	Definition
^MEAS^Centreline_LENGTH_	Length of the ROI centreline (an inversed U-shaped line following the centre of the myocardium) measured in cm from left basal ROI point, along the ROI centreline to the right basal ROI point.
^MEAS^LV_LENGTH_	LV length measured in cm from the apical ROI centreline point to the centre of the line between the two basal ROI points.
^MEAS^LV_WIDTH_	Average ROI width in mm.
^MEAS^APICAL_VER_	Vertical distance from the transducer to the apical ROI centreline point in mm.
^MEAS^APICAL_HOR_	Horizontal deviation from mid of recording to the apical ROI centreline point in mm, where negative values indicate leftward deviation and positive values indicate rightward deviation.
^MEAS^BASE_VER_	Vertical distance from the transducer to the left and right basal ROI points in mm.
^MEAS^Knots	Defined as the number of identification points (knots) placed on the endocardial border by the operator to initialize the ROI during strain analyses.
^MEAS^LV_FORESHORTENING_	Systolic vertical movement of the apical ROI centreline point in mm. Positive values indicate foreshortening (downward movement in image), while upward movement in the image during systole is provided as negative values.

The explanations for the measurement procedure specific (abbreviations) are provided under ‘Definition’ (right column). ^MEAS^LV_FORESHORTENING_ was calculated from the position of the apical ROI centreline point at end-diastole and end-systole, while the others were directly extracted from the 2D strain software metadata. Abbreviation: 2D, two-dimensional; ROI, region of interest.

### Statistical methods

We assessed the data using histograms and quantile–quantile plots to determine the distributions. The normally distributed variables are presented as means with standard deviations (SD). Proportions are presented by numbers (*n*) and percentages. The normal reference range for LV GLS was presented as the 95% confidence interval (mean − 1.96 SD, mean + 1.96 SD) as we expected 95% of the normal population to fall within this interval. Descriptive data of the distribution of the quality indicators for image recordings were shown in histograms, with counts plotted against the size of the quality indicator.

Comparisons between sexes were performed by Student’s *t*-test for independent samples. We used general linear models to study the associations of LV GLS with recording-specific and GLS measurement procedure-specific parameters. As we have recently shown the GLS to be associated with subject-specific characteristics, we performed both unadjusted and adjusted analyses. Thus, the analyses using recording-specific quality indicators ^REC^APICAL_VER_, ^REC^APICAL_HOR_, ^REC^LV_FORESHORTENING_, and ^REC^LV_LENGTH_ were adjusted for age, sex, and body surface area calculated by the formula by DuBois. Analyses evaluating rotation and tilt parameters were not adjusted, while the analyses using the measurement procedure-specific quality indicators ^MEAS^LV_LENGTH_ and ^MEAS^Centreline_LENGTH_ were adjusted for age, sex, and body surface area, while analyses using ^MEAS^APICAL_VER_, ^MEAS^APICAL_HOR_, ^MEAS^BASE_VER_, and ^MEAS^LV_FORESHORTENING_ were adjusted for age, systolic blood pressure, and mean left ventricular wall thickness. Further, analyses using ^MEAS^LV_WIDTH_ were adjusted for age, systolic blood pressure, and mean left ventricular wall thickness. Finally, ^MEAS^Knots was adjusted for body mass index and image quality score. The decision on which parameter to adjust for was predefined based on clinical experience and known associations between GLS and subject characteristics.^8^ The average values from the two paired operators were used when evaluating the association of LV GLS with recording-specific characteristics.

Moreover, GLS and measurement-specific differences between the experts and the experienced or intermediate experienced operator groups were calculated. Here, a positive beta coefficient (*β*) indicated higher absolute GLS in % per unit difference for the specified parameter. GLS is presented as absolute values for descriptive values when presenting differences between groups and associations with specific characteristics. All analyses were performed using SPSS Statistics, version 29 (IBM Corporation, Armonk, NY, USA). A two-sided *P*-value <0.05 was considered statistically significant.

## Ethical approval

This project was approved by the Mid-Norway Regional Committee for Medical and Health Research Ethics (REK 7160). All parts of the study were performed in compliance with the ethical principles in the Declaration of Helsinki. A Data Protection Impact Assessment (DPIA) was conducted and approved by the institutional personal data officer at St. Olavs Hospital and the Norwegian University of Science and Technology.

## Results

### Population

Comprehensive details of the healthy study population are previously published.^8,16^ These data are summarized in the Methods section (*Table 1*). Age, systolic blood pressure and body mass index was mean (SD) 58^12^ years, 128^18^ mm Hg, and 25.8 (3.6) kg/m^2^, respectively.

### GLS measurements

The overview of GLS measurements and their relations to subject-specific characteristics has been previously published.^8^ In summary, the main results showed that normal GLS by the 18-segments model ranged from 15.6% to 23.9%.^8^ GLS was lower in men and with higher age. Females had an average of 1%-point higher GLS than men. GLS was associated with age, and 20 years higher age corresponded to approximately 1%-point lower GLS.

### Recording-specific quality indicators and GLS

**Figure 4:**
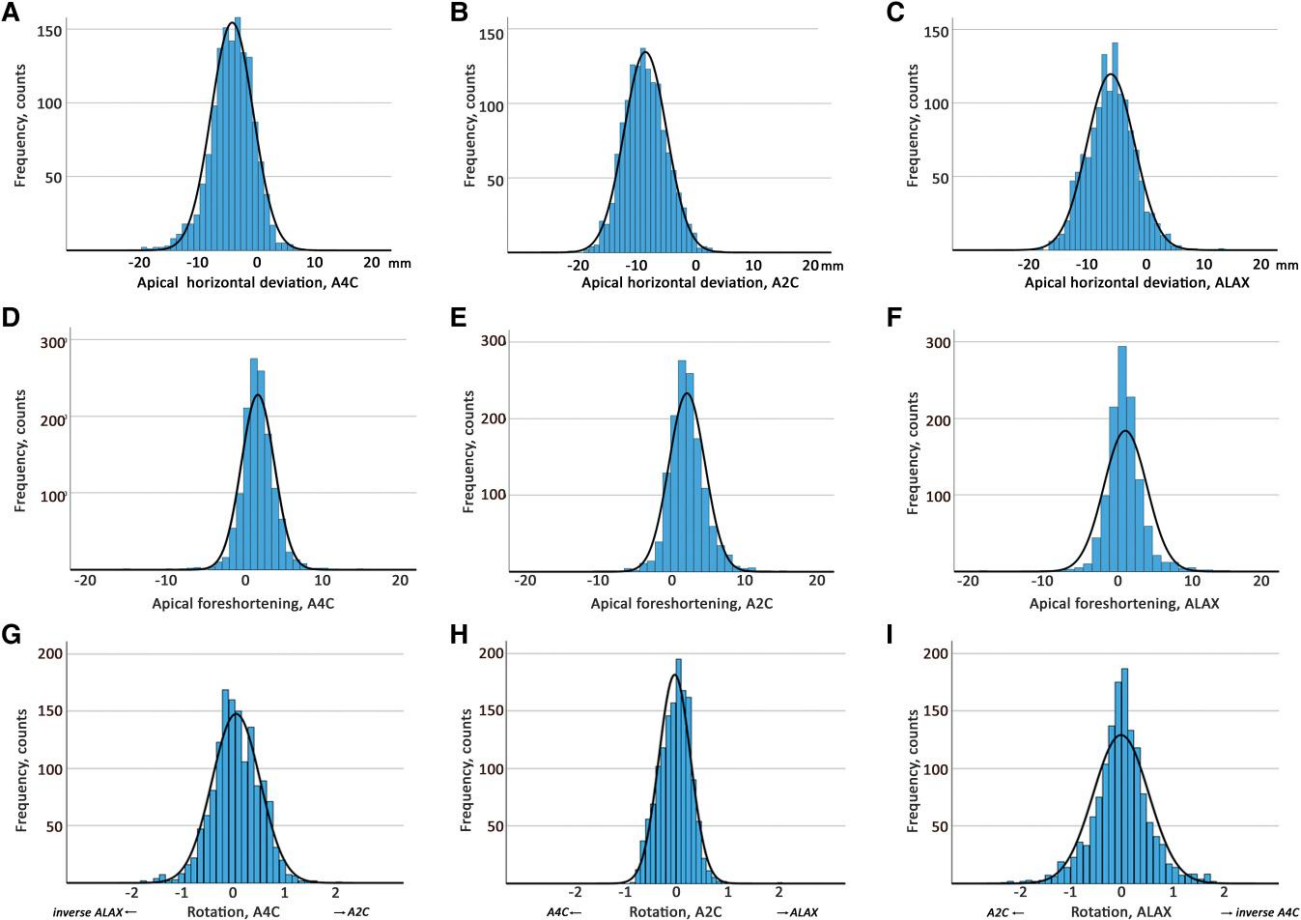
Distribution of image recording specific characteristics. Histograms showing the distribution of the apical horizontal deviation (*A–C*), where negative values indicate leftward orientation of the apex. *D–F* show the apical foreshortening, where positive values indicate downward movement of the apical endocardial point in systole, whereas negative values indicate that the apex moves towards the probe in systole. *G–I* show the rotational alignment of the cut-plane compared to an optimal cut-plane as defined by the rotation and tilt software. A score of 0 indicates perfect alignment, while negative values indicate clockwise rotation of the transducer.

*Figure 4* presents histograms and normal distribution curves of selected recording-specific characteristics (apical horizontal deviation, apical foreshortening, and rotational alignment). The extracted data showed that the apex was positioned slightly to the left of the centre of the recording, illustrated by the location of the peak of distributions between −4 mm and −9 mm (A-C). Apical LV foreshortening was limited (D-F), being ≤2.1 mm per view and mean (SD) 1.8 (1.7) mm globally. Rotational and tilting alignment to the preferred cut-plane was well standardized (G-I) with overall mean (SD) −0.00 (0.31) and −0.05 (0.74) on the DL-software-specific relative scale, respectively. Furthermore, just 141 (10.0%) subjects had rotational alignment scores beyond ±0.5. The findings were consistent across the three standard apical views. The distribution of data overall and by each of the three standard apical views are shown in Supplementary data online, *Table S1*.

The associations of GLS with image recording-specific characteristics are presented in *Table 5* as averages for the three standard apical views. In short, GLS was lower with reduced image quality and with increased deviations from the optimal rotational alignment. In analyses adjusted for age, sex and BSA, foreshortened LV recordings were associated with higher GLS (0.8%-points per cm LV foreshortening, *P* = 0.01).

**Table 5:** The associations of GLS with image recording-specific characteristics

Parameters (*n* ≥ 1357 (96%))	Unadjusted *β* (95% CI)	*P*_unadj._	Adjusted β (95% CI)	*P_adj._*
^a^Image quality reduced	−0.84 (−1.08, -0.59)	<0.001	—	—
^b^Vertical position—apex, ED, mm	−0.03 (−0.05, -0.01)	0.02	0.00 (−0.02, 0.03)	0.77
^b^Horizontal deviation—apex, ED, mm^c^	0.04 (−0.01, 0.09)	0.12	−0.00 (−0.05, 0.04)	0.92
^b^LV length, ED, mm	−0.02 (−0.03, −0.01)	0.003	−0.01 (−0.03, 0.01)	0.19
^b^Syst. apical foreshortening, mm	0.04 (−0.03, 0.11)	0.24	0.08 (0.02, 0.12)	0.01
^a^Rotational alignment	−0.67 (−1.24, −0.11)	0.02	—	—
^a^Tilt alignment	−0.21 (−0.44, 0.02)	0.08	—	—

Regression based on absolute values for LV GLS. Analyses adjusted for:

^a^No adjustment performed.

^b^Age, sex and body surface area.

^c^Negative values leftward for centreline, positive values rightward.

Specific data for the three standard apical views are presented in Supplementary data online, *Table S2*. For the associations of GLS with horizontal deviation of the apical points from the mid of the sector, there were positive associations for A4C and A2C (both 0.09%-points per mm), while there was a negative association for ALAX (−0.04%-points per mm). For the associations of rotational alignment with the preferred cut-plane and GLS, the findings differed across the three apical views, with a negative association for A4C, a positive association for A2C, and no significant association for ALAX. Furthermore, a tilting misalignment with the preferred cut-plane showed consistent negative associations across apical views, though not significant for A4C.

### Measurement procedure characteristics and GLS

*Table 6* shows the specific parameters extracted from the strain measurement procedures. Absolute GLS values were lower with larger averaged ROI width (0.76%-points per mm larger ROI width, *P* < 0.001). Apical foreshortening as defined by downward motion of the apical ROI points during systole was associated with higher absolute GLS (0.54%-points per mm systolic foreshortening, *P* < 0.001). These associations had the same direction when adjusting averaged ROI width for age, systolic blood pressure, and mean left ventricular wall thickness and when adjusting apical systolic foreshortening for body mass index. In short, in the unadjusted analysis, only the vertical position of the apex and the apical horizontal deviation had no significant effect on the strain value. Placing more knots (identification points) on the endocardial border to initialize the ROI and apical systolic foreshortening were both associated with higher GLS. When adjusting for age, sex, and body surface area, we found that a 1 cm larger LV length was associated with 0.2%-points higher GLS (*P* < 0.001), as opposed to a 0.09%-points (*P* = 0.02) drop in the unadjusted analysis. The detailed results for the standard apical views were well aligned to the results for the averaged values (see Supplementary data online, *Table S3*-*S4*).

**Table 6:** Associations of global longitudinal strain with specific measurement procedure parameters

Parameters (*n* ≥ 2738 (97%))	Unadjusted *β* (95% CI)	*P*_unadj._	Adjusted *β* (95% CI)	*P_adj._*
^a^ROI centreline length, ED, cm	−0.06 (−0.11, −0.01)	0.028	0.09 (0.02, 0.16)	0.017
^a^LV length by ROI, ED, cm	−0.09 (−0.17, −0.01)	0.02	0.21 (0.10, 0.32)	<0.001
^b^Averaged ROI width, ED, mm	−0.76 (−0.83, −0.69)	<0.001	−0.66 (−0.73, −0.58)	<0.001
^c^Vertical position—apex, ED, mm	−0.00 (−0.02, 0.01)	0.59	−0.07 (−0.10, −0.04)	<0.001
^c^Horizontal deviation—apex, ED, mm^d^	−0.03 (−0.06, 0.00)	0.082	−0.02 (−0.05, 0.01)	0.114
^c^Vertical position—base, ED, mm	−0.01 (−0.19, −0.00)	0.014	−0.00 (−0.01, 0.01)	0.56
^e^Number of ROI knots, *n*	0.10 (0.06, 0.14)	<0.001	0.07 (0.02, 0.11)	0.004
^c^Syst. apical foreshortening, mm	0.54 (0.47, 0.61)	<0.001	0.71 (0.65, 0.78)	<0.001

Analyses adjusted for:

^a^Age, sex and body surface area.

^b^Age, systolic blood pressure and mean left ventricular wall thickness.

^c^Body mass index.

^d^Mean (SD), with negative values leftward for centreline, positive values rightward. Regression based on absolute values.

^e^Body mass index and image quality score.

Abbreviations: CI, confidence interval; ED, end-diastolic; LV, left ventricular; ROI, region of interest; SD, standard deviation.

*Table 7* shows the associations between operators’ differences in repeated GLS measurement procedures within subjects and variations in specific parameters from strain measurement procedures. In short, these results followed the same pattern as described above. ^MEAS^Centreline_LENGTH_ and ^MEAS^LV_FORESHORTENING_ were significantly associated with GLS, corresponding to 1%-point higher GLS by 1 cm shorter ^MEAS^Centreline_LENGTH_ and 1 mm ^MEAS^LV_FORESHORTENING_ (both *P* < 0.001). In other words, based on repeated strain measurements from the same recording, a 1 cm shorter centreline of the entire inverse U-shaped myocardial ROI or 1 mm downward systolic motion of the apical ROI corresponded to 1%-point higher GLS. Adjustment of these analyses did not significantly alter the results. Even though being statistically significant, differences in ^MEAS^LV_WIDTH_ and positioning of basal and apical points influenced GLS less than the ^MEAS^Centreline_LENGTH_ and ^MEAS^LV_FORESHORTENING_. The view-specific results for the three standard apical views were well aligned with the results for the averaged values (see Supplementary data online, *Table S5*-*S6*).

**Table 7:** Associations of differences in global longitudinal strain across repeated analyses within subjects with differences in measurement procedure-specific parameters

Parameters (*n* ≥ 1333 (94%))	Unadjusted *β* (95% CI)	*P*_unadj._	Adjusted *β* (95% CI)	*P_adj._*
^a^ROI centreline length, ED, cm	−0.98 (−1.14, −0.82)	<0.001	−0.87 (−1.02, −0.71)	<0.001
^a^LV length by ROI, ED, cm	0.32 (−0.17, 0.82)	0.20	0.47 (−0.01, 0.96)	0.056
^b^Averaged ROI width, ED, mm	−0.17 (−0.27, −0.08)	<0.001	−0.19 (−0.28, −0.10)	<0.001
^c^Vertical position—apex, ED, mm	0.48 (0.43, 0.54)	<0.001	0.48 (0.42, 0.53)	<0.001
^c^Horizontal deviation—apex, ED, mm^d^	0.08 (0.04, 0.12)	<0.001	0.08 (0.04, 0.13)	<0.001
^c^Vertical position—base, ED, mm	−0.03 (−0.08, 0.02)	0.18	−0.04 (−0.09, 0.01)	0.100
^e^Number of ROI knots, *n*	−0.16 (−0.48, 0.15)	0.32	−0.09 (−0.42, 0.25)	0.603
^c^Systolic foreshortening—apex, mm	1.26 (1.14, 1.39)	<0.001	1.20 (1.07, 1.33)	<0.001

GLS and measurement procedure specific differences were processed as data from the expert minus data from the experienced or intermediate experienced operators. Positive *β* indicate higher GLS in % per unit difference in the specified parameters between the repeated analyses. Analyses adjusted for:

^a^ Age, sex and body surface area.

^b^ Age, systolic blood pressure and mean left ventricular wall thickness.

^c^Body mass index.

^d^Mean (SD), with negative values leftward for centreline, positive values rightward. Regression based on absolute values.

^e^Body mass index and image quality score.

Abbreviations as in *Table 6*.

## Discussion

In this large population-based echocardiographic study, we focused on novel, automatically extracted markers reflecting the standardization and quality of both the recorded echocardiograms and the measurement procedure. Additionally, we examined the relations of these quality indicators with the GLS measurements. Shortly, apical LV recordings were well standardized and LV systolic apical foreshortening was below 2 mm. In unadjusted analyses GLS was lower with reduced image quality, longer LVs, wider ROIs during the GLS measurement procedures, and deeper position of the LV apex in the sector. Moreover, GLS was 0.7% higher per mm LV apical foreshortening, and GLS was higher when more knots were placed under initialization of the ROI during GLS analysis.

### Population

The study population was recruited from a healthy subsample, and subjects with pathological findings on echocardiography such as valvular disease and LV dysfunction were excluded.

The associations of lower LV GLS with higher age and male sex have been shown previously.^18^ However, these associations have not been consistent across different studies.^7,19–21^ Possible explanations may relate to the distributions of age and sex, cohort sizes, as well as differences in the recorded images and measurement procedures. The presented population was approximately 10 years older compared to the World Alliance Societies of Echocardiography (WASE) and European Association of CardioVascular Imaging Normal Reference Ranges for Echocardiography (NORRE) studies.^7,21^ A recently published meta-analysis indicated that most normal LV GLS data originated from samples of young subjects, which may challenge the comparison between studies.^22^

Even though some differences exist for LV GLS between studies, we expect that the associations of quality indicators extracted from recordings and measurement procedures are more general. Thus, the exact numbers presented for the influence of recording- and measurement-specific quality indicators on LV GLS may vary between populations and operators; it is expected that the principal findings of our study can be generalized to others.

### Automated evaluation of quality in echocardiographic data

Previously, it has been shown that tracking quality by a software-specific block-matching algorithm is best in the septal and inferior walls.^19^ Also, the importance of LV apical foreshortening on GLS measurements has been established.^10^ By comparing foreshortened apical recordings corresponding to one rib-space higher position of the transducer than the optimal, the latter study found 8–14% relative overestimation of mid-wall GLS by two different GLS software. Compared to our study, the study evaluating a block-matching algorithm relates to the finding of lower GLS with lower subjective image quality score, while the latter study relates to the finding of higher GLS when the recordings or the ROI during the measurement procedure are foreshortened.

In this context, we have recently presented a more detailed image quality score considering aspects from view alignment, apical position, identification of the endocardial border and mitral annulus, which differentiated well with respect to operators’ level of experience.^11,12^ However, the scope of the previous work was to evaluate the importance of image quality for heart failure diagnostics by hand-held ultrasound diagnostics, and no associations with GLS were evaluated. Importantly, image quality characteristics influenced the feasibility and reliability of automated estimation of LV ejection fraction.^11^

The introduction of DL tools in echocardiography allows for both real-time image analysis and guiding of the operator to achieve optimal recordings. Several studies have shown that operators may improve standardization of the recording when using DL tools for real-time guiding during echocardiographic scanning.^13,14,17,23^ Among novice operators, real-time guiding most often resulted in images of diagnostic quality.^24,25^ Using the same software as used in the present study, experienced sonographers optimized and standardized their recordings by significantly reducing LV apical foreshortening and misalignment from the preferred cut-planes for rotation and tilting.^17,23^ Thus, these studies indicate that there is variability also in echocardiographic databases from well-recognized study groups, and it is possible to extract quality indicators from recorded echocardiograms using deep learning.

### The importance of recordings and measurement procedures for GLS

Baron and co-authors^26^ showed that for the reproducibility of LV ejection fraction, the effect of the measurement procedure was more important than the recordings, while for LV GLS the effect of the recordings was more important than the measurement procedures. In a recent publication evaluating experienced operators, we found that the measurement procedures were more important than foreshortening of the LV recordings for the variability of LV ejection fraction and GLS.^17^ Even though many characteristics of the measurement procedure significantly influenced the GLS measurements in repeated analyses in the present study, the within-pair differences in length of the ROI and systolic apical ROI foreshortening were most important. However, as recently shown, the differences between the two groups of operators analysing GLS were small.^8^ The consistent findings of the presented associations of quality indicators and GLS within and between operators make the findings trustworthy.

### Clinical relevance

This study shows that several factors characterizing echocardiographic recordings and the strain measurement procedure influence the size and robustness of the measurement. Even though several of the proposed quality indicators are non-standard and rarely included in the clinical reports, they are important to describe the trustworthiness of the outcome. For example, foreshortening of the LV in the recording (corresponding to ^REC^LV_FORESHORTENING_) and systolic foreshortening of the ROI during analysis, i.e. downward systolic motion of the apical points of interest (corresponding to ^MEAS^LV_FORESHORTENING_) was associated with higher strain measurements. These findings extend the literature base suggesting that any degree of foreshortening influence is important for precision and reproducibility of strain measurements.^10^ Importantly, the same characteristics extracted from recordings and measurement procedures shown to associate with the size of the strain measurements were also important for the reproducibility between the operators. Thus, putting emphasis on the quality of recordings and measurement procedures may improve the specificity and reproducibility of echocardiographic strain measurements. The automated extraction of such quality indicators by DL and software metadata may be a feasible way to advance clinical evaluation of quality in echocardiography.

### Strength and limitations

Major strengths relate to the stringent methodology and the large study population, strongly indicating that the presented findings are actual associations. Further, the DL software used in this work have been validated with good agreement towards expert operators with respect to reference measurement of LV length and apical foreshortening,^17^ as well as tilt and rotation across the three standard apical views.^23^ Even though data for alignment of tilt and rotation with the preferred cut-planes are presented both as averaged and view-specific values, it is important that these indices must be interpreted on the view-specific level.

Importantly, the DL software provided automatically extracted quality indicators that can be easily overseen by human experts, as shown for the DL provided measurements of LV apical and basal positions as well as the calculations of LV length and apical LV foreshortening as shown in Supplementary data online, *Figure S1*. Implementing such a level of transparency and interpretability may improve clinicians' trust in DL-based technology, in contrast to end-to-end (‘black box’) approaches.

The study also has some limitations. Excluding subjects with cardiac disease may reduce the generalizability to populations with heart diseases and reduced LV function. All procedures were performed by well-experienced operators, and other indices could potentially be more important among less experienced operators. Even though the echocardiograms were acquired using high-end scanners, it is unknown whether advances in ultrasound technology since 2017, such as improved image quality, would interfere with the strain measurements and the suggested quality indicators. The presented values relate both to the population studied, the operators and equipment included, as well as the recording and measurement-specific characteristics. Thus, care must be taken when generalizing to other settings. Lastly, the DL-based software for rotation and tilt alignment is based on training on echocardiograms from populations where a clinical expert defined the optimal 2D cut-planes derived from 3D recordings. Importantly, the tools presented in this work may allow for revitalization of the discussion of optimizing cut-planes. As shown previously, using the rotation alignment tool it will not always be 120 degrees between the A4C and the ALAX views.^13^ However, DL tools such as the ones presented here may be trained according to future expert consensus and allow for assessment of quality indicators across studies.

## Conclusions

Using novel DL-based software and strain-specific metadata, we present relevant quality indicators of echocardiographic recordings and measurement procedures and highlight their associations with GLS. Both the echocardiographic recordings and measurement procedures were well standardized, absolute GLS was lower with less apical LV foreshortening, longer LVs, deeper position of the LV apex. Thus, objective evaluation of the quality of recordings and measurement procedures provides an opportunity for quality assessment of echocardiographic studies which may aid scientists in interpretation of study-specific findings.

## Supplementary Material

qyag124_Supplementary_Data

## Data Availability

Data from this study may be made available upon reasonable request to the corresponding author.

## Lead author biography

Even Olav Jakobsen, RN, MSc, works as a clinical and scientific sonographer at St. Olavs hospital and Norwegian University of Science and Technology, Trondheim, Norway. He is experienced in advanced echocardiographic methods and have co-authored several manuscripts within the field, also including the use of artificial intelligence to optimize echocardiographic recordings and measurements.
