# Supplementary material for: Automated quality indicators for echocardiographic recordings and measurements: consequences for left ventricular global longitudinal strain
Source: Eur Heart J Imaging Methods Pract. 2026 Jul 20;4(3):qyag124. doi: 10.1093/ehjimp/qyag124 (PMC13398993; doi:10.1093/ehjimp/qyag124)
Supplement: qyag124_Supplementary_Data [file qyag124_supplementary_data.docx]

**Supplementary method.**

### *Echocardiographic recordings*

Echocardiographic recordings were performed according to recommendations from the European Association of Cardiovascular Imaging (EACVI) and the American Society of Echocardiography (ASE)(1). All echocardiograms were acquired by two experienced sonographers (KS and EOJ) (>2,000 recordings and readings each) at the study center located at Levanger Hospital, Levanger, Norway. The images were captured using Vivid E95 scanners (GE Healthcare, Horten, Norway) with M5Sc-D and 4VD phased-array transducers. At least three cardiac cycles were recorded per view and guideline-directed dedicated recordings were used for all four cardiac chambers. For LV strain this corresponded to apical 4-chamber (A4C), 2-chamber (A2C) and long-axis (ALAX) views. Frame rate ranged from 71 to 85 Hz. In addition, dedicated views of the other chambers, blood flow Doppler and tissue Doppler images were recorded in all subjects. Images were optimized by quiet breathing or breath-hold. Data were digitally stored and analyzed retrospectively.

### *Strain measurements*

Strain was analyzed using the 2D strain speckle-tracking software (2DS) in EchoPAC SWO version 204 (GE Healthcare) by four experienced operators affiliated with the EACVI accredited echocardiographic laboratory at St. Olavs University Hospital, Trondheim, Norway (JN, EOJ, BG and HD). The level of expertise and number of analyses for each of the four operators processing the GLS measurements are presented in Table 2. Each operator chose one of three recorded cycles from each of the A4C, A2C and ALAX views for the measurement of GLS. First, GLS was processed by one cardiology resident (JN) or one experienced sonographer (EOJ). All view-specific strains were then re-read and adjusted by one of two cardiologist experts in echocardiography and strain imaging (BG and HD). The ROI initializations from the initial analyses were then used, adjusted or replaced to achieve the best possible tracking of the myocardial motion.

The ROI were placed to include the whole myocardium, but excluding the pericardium, as well as any trabeculae and the papillary muscles. Some degree of manual adjustments of the semi-automatically segmentation initialized by the software were deemed necessary in all recordings. LV strains were analyzed in 18 myocardial segments, i.e. six segments per view. Segmental strain values were excluded by the operators if the tracking was suboptimal and the provided segmental strain value influenced GLS. This process was left to the decision of the operators. Individual segments with reduced tracking were included if the operator deemed the whole wall segmental strain to be acceptable. Only subjects with at least 10 out of 18 segments accepted were included in the reference ranges. The quality of the recorded views was subjectively assessed by two operators (JN and EOJ). The images were categorized as being of reduced quality or not. The timing of end-systole (ES) was defined by the aortic valve closure visually assessed in the ALAX view and thereafter used for A4C and A2C. If the aortic valve could not be visualized, the default ES timing or Doppler method was used. Timing of end-diastole (ED) was defined by the 2DS software default value and adjusted using the timing of the mitral valve closure in the ALAX view if necessary. Default settings were used for drift compensation, as well as for temporal and spatial smoothing.

During measurement of GLS, the provided values and metadata were captured with information about the placement of the region of interest (ROI) and the strain trace for the whole cardiac cycle in every image plane.

1. Lang RM, Badano LP, Mor-Avi V et al. Recommendations for cardiac chamber quantification by echocardiography in adults: an update from the American Society of Echocardiography and the European Association of Cardiovascular Imaging. Journal of the American Society of Echocardiography : official publication of the American Society of Echocardiography 2015;28:1-39 e14.

**Supplementary Figure S1.** Examples of deep learning measurements of landmark positions and left ventricular length overseen by experts.

**
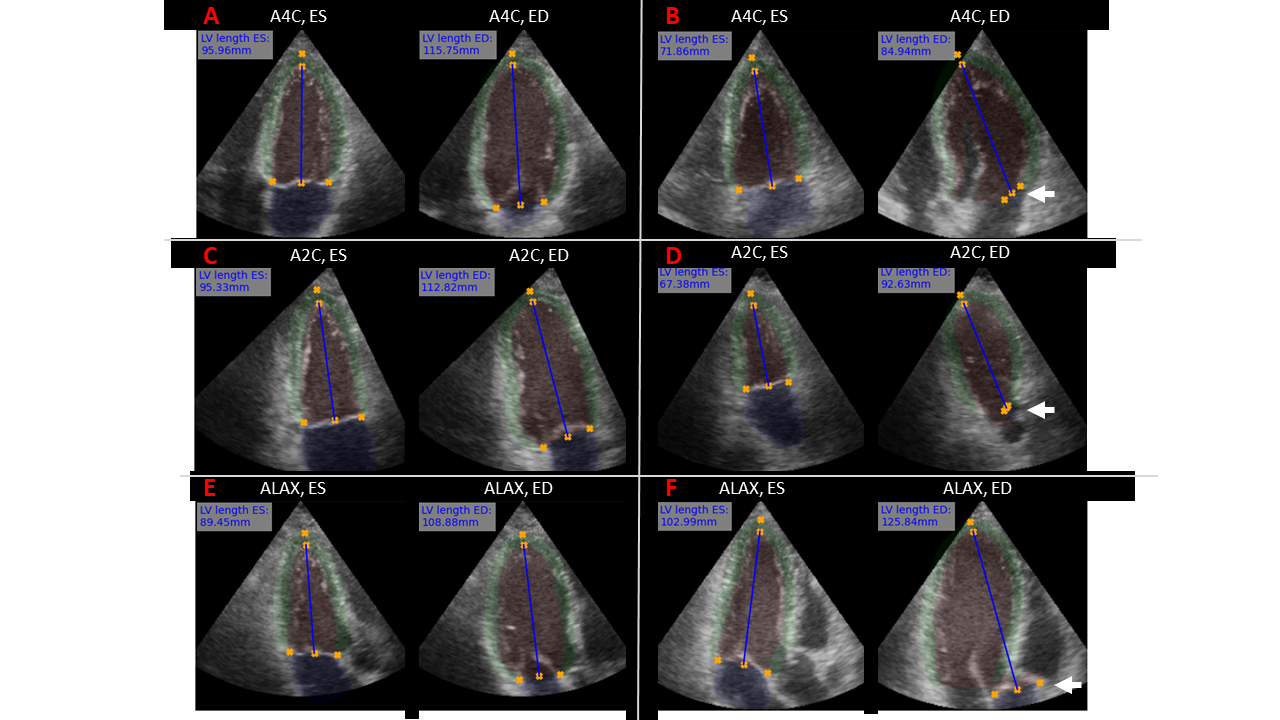
**

*Supplementary Figure S1. Landmark points at the apical endocardium and epicardium and three mitral annular points (left, right and mid) all marked by boxes. The LV length estimate is shown by the line from apical endocardial point to the mid of the mitral annular line. Left panels shows examples accepted by the human experts for A4C, A2C and ALAX (A, C and E), whereas right panels show examples rejected by the human experts where the segmentation failure is indicated by the arrows (B, D and F). Abbreviations: A4C, apical 4-chamber view; A2C, apical 2-chamber view; ALAX, apical long-axis view; ED, end-diastole; ES, end-systole, LV, left ventricle.*

**Supplementary Table S1.** Distribution of recording specific characteristics.

| **Parameters** | **Average of views**  **Mean (SD)** | **A4C**  **Mean (SD)** | **A2C**  **Mean (SD)** | **ALAX**  **Mean (SD)** |
| --- | --- | --- | --- | --- |
| Numbers (minimum) | 1391 | 1335 | 1358 | 1144 |
| ^a^Image quality reduced | 1.28 (0.45) | - | - | - |
| ^b^Vertical position - apex, ED, mm | 26.2 (4.8) | 25.9 (5.1) | 25.9 (4.9) | 26.9 (5.7) |
| ^b^Horizontal deviation - apex, ED, mm* | -6.5 (2.3) | -4.4 (3.6) | -9.1 (3.7) | -5.7 (4.1) |
| ^b^LV length, ED, mm | 90 (8) | 90 (8) | 90 (9) | 91 (9) |
| ^b^Syst. apical foreshortening, mm | 1.8 (1.7) | 1.9 (2.1) | 2.1 (2.5) | 1.2 (2.9) |
| ^a^Rotational alignment | -0.00 (0.31) | 0.02 (0.48) | -0.02 (0.31) | -0.01 (0.54) |
| ^a^Tilt alignment | -0.05 (0.74) | -0.01 (1.06) | -0.04 (1.29) | -0.10 (1.17) |

*Supplementary Table S1. Lowest available numbers were for the systolic apical foreshortening parameter. *Negative values leftward for centerline, positive values rightward.* *Abbreviations: A4C, apical 4-chamber view; A2C, apical 2-chamber view; ALAX, apical long-axis view; ED, end-diastole; SD, standard deviation.*

**Supplementary Table S2.** Associations of left ventricular regional longitudinal strain from standard apical views with specific image recording parameters.

| **Parameters** | **Unadjusted β (95% CI)** | | | | ***P*_unadj._** | | **Adjusted β (95% CI)** | ***P_adj._*** |  |
| --- | --- | --- | --- | --- | --- | --- | --- | --- | --- |
| **Apical 4-chamber view (N≥1,323 (94%))** | | | | | | | | | |
| ^b^Vertical position - apex, ED, mm | -0.02 (-0.05, 0.00) | | | | 0.08 | | 0.01 (-0.02, 0.03) | 0.52 |  |
| ^b^Horizontal deviation - apex, ED, mm* | 0.09 (0.06, 0.13) | | | | <0.001 | | 0.06 (0.02, 0.09) | 0.001 |  |
| ^b^LV length, ED, mm | -0.02 (-0.04, -0.01) | | | | 0.003 | | -0.01 (-0.04, 0.01) | 0.17 |  |
| ^b^Syst. apical foreshortening, mm | 0.08 (0.02, 0.15) | | | | 0.006 | | 0.10 (0.05, 0.16) | <0.001 |  |
| ^a^Rotational alignment | -0.51 (-0.94, -0.08) | | | | 0.02 | | - | - |  |
| ^a^Tilt alignment | -0.14 (-0.34, 0.07) | | | | 0.18 | | - | - |  |
| **Apical 2-chamber view (N≥1,341 (95%))** | | | | | | | | |  |
| ^b^Vertical position - apex, ED, mm | | -0.02 (-0.05, 0.01) | | 0.16 | | 0.03 (-0.00, 0.06) | | 0.09 |  |
| ^b^Horizontal deviation - apex, ED, mm* | | 0.09 (0.05, 0.12) | | <0.001 | | 0.06 (0.03, 0.10) | | <0.001 |  |
| ^b^LV length, ED, mm | | -0.01 (-0.02, 0.01) | | 0.32 | | 0.00 (-0.02, 0.02) | | 0.83 |  |
| ^b^Syst. apical foreshortening, mm | | 0.06 (0.00, 0.12) | | <0.001 | | 0.06 (0.01, 0.12) | | 0.02 |  |
| ^a^Rotational alignment | | 0.86 (0.11, 1.60) | | 0.02 | | - | | - |  |
| ^a^Tilt alignment | | -0.19 (-0.37, -0.02) | | 0.03 | | - | | - |  |
| **Apical long-axis view (N≥1,131 (80%))** | | | | | | | | |  |
| ^b^Vertical position - apex, ED, mm | | | -0.04 (-0.06, -0.01) | | 0.002 | | -0.02 (-0.04, 0.01) | 0.15 |  |
| ^b^Horizontal deviation - apex, ED, mm* | | | -0.04 (-0.07, -0.01) | | 0.02 | | -0.04 (-0.07, -0.00) | 0.03 |  |
| ^b^LV length, ED, mm | | | -0.02 (-0.04, -0.00) | | 0.02 | | -0.01 (-0.03, 0.01) | 0.45 |  |
| ^b^Syst. apical foreshortening, mm | | | 0.03 (-0.02, 0.08) | | 0.22 | | 0.05 (0.01, 0.10) | 0.03 |  |
| ^a^Rotational alignment | | | -0.25 (-0.62, 0.12) | | 0.19 | | - | - |  |
| ^a^Tilt alignment | | | -0.32 (-0.50, -0.15) | | <0.001 | | - | - |  |

*Supplementary Table S2. Abbreviations: β, beta coefficient; CI, confidence interval, otherwise as in Supplementary Table S1. For the horizontal deviation analyses, there were no significant changes when using apical centerline points instead of the apical endocardial points in the analyses (data not shown).*

**Supplementary Table S3.** Distribution of specific strain measurement procedure parameters.

| **Parameters** | **Average of views**  **Mean (SD)** | **A4C**  **Mean (SD)** | **A2C**  **Mean (SD)** | **ALAX**  **Mean (SD)** |
| --- | --- | --- | --- | --- |
| ^a^ROI centerline length, ED, cm | 18.9 (16.4) | 19.4 (1.8) | 19.7 (1.8) | 17.7 (1,7) |
| ^a^LV length by ROI, ED, cm | 10.9 (1.1) | 11.1 (1.1) | 11.2 (1.1) | 10.4 (1.1) |
| ^b^Averaged ROI width, ED, mm | 7.1 (1.1) | 7.0 (1.0) | 7.3 (1.1) | 7.2 (1.2) |
| ^c^Vertical position - apex, ED, mm | 24.1 (6.2) | 23.8 (6.3) | 23.4 (6.4) | 24.9 (6.6) |
| ^c^Horizontal deviation - apex, ED, mm* | -1.3 (3.7) | 0.9 (5.6) | -5.3 (6.8) | 0.4 (6.9) |
| ^c^Vertical position - base, ED, mm | 105 (1.0) | 108 (10) | 108 (10) | 100 (1.0) |
| ^d^Number of ROI knots, *N* | 11 (2.0) | 11.6 (2.1) | 11.0 (2.1) | 10 (2.1) |
| ^c^Syst. apical foreshortening, mm | 1.8 (1.2) | 1.8 (1.6) | 1.9 (1.6) | 1.8 (1.4) |

*Supplementary Table S3. All parameters available in at least 96%. Abbreviations: As in Supplementary Table S1.*

**Supplementary Table S4.** Associations of left ventricular regional longitudinal strain from standard apical views with specific strain measurement procedure parameters.

| **Parameters** | **Unadjusted β (95% CI)** | | ***P*_unadj._** | **Adjusted β (95% CI)** | | | ***P_adj._*** |  |  |
| --- | --- | --- | --- | --- | --- | --- | --- | --- | --- |
| **Apical 4-chamber view (N≥2,731 (97%))** | | | | | | | |  |  |
| ^a^ROI centerline length, ED, cm | -0.10 (-0.15, -0.04) | | <0.001 | 0.02 (-0.06, 0.09) | | | 0.64 |  |  |
| ^a^LV length by ROI, ED, cm | -0.13 (-0.21, -0.04) | | 0.003 | 0.10 (-0.02, 0.23) | | | 0.11 |  |  |
| ^b^Averaged ROI width, ED, mm | -0.80 (-0.89, -0.71) | | <0.001 | -0.70 (-0.78, -0.61) | | | <0.001 |  |  |
| ^c^Vertical position - apex, ED, mm | 0.00 (-0.01, 0.01) | | 0.90 | 0.03 (0.01, 0.05) | | | 0.01 |  |  |
| ^c^Horizontal deviation - apex, ED, mm* | -0.02 (-0.05, 0.00) | | 0.10 | -0.02 (-0.05, 0.01) | | | 0.12 |  |  |
| ^c^Vertical position - base, ED, mm | -0.02 (-0.02, -0.01) | | 0.001 | -0.01 ( -0.02, -0.00) | | | 0.03 |  |  |
| ^d^Number of ROI knots, *N* | 0.06 (0.01, 0.10) | | 0.01 | 0.04 (-0.01, 0.09) | | | 0.09 |  |  |
| ^c^Syst. apical foreshortening, mm | 0.50 (0.43, 0.57) | | <0.001 | 0.59 (0.53, 0.66) | | | <0.001 |  |  |
| **Apical 2-chamber view (N≥2,719 (96%))** | | | | | | | | | |
| ^a^ROI centerline length, ED, cm | | -0.04 (-0.09, -0.02) | 0.2 | | 0.05 (-0.03, 0.13) | 0.19 | | |  |
| ^a^LV length by ROI, ED, cm | | -0.01 (-0.10, -0.09) | 0.89 | | 0.40 (0.26, 0.54) | <0.001 | | |  |
| ^b^Averaged ROI width, ED, mm | | -0.76 (-0.85, -0.67) | <0.001 | | -0.66 (-0.74, -0.57) | <0.001 | | |  |
| ^c^Vertical position - apex, ED, mm | | 0.01 (-0.00, 0.03) | 0.14 | | 0.05 (0.03, 0.07) | <0.001 | | |  |
| ^c^Horizontal deviation - apex, ED, mm* | | -0.08 (-0.10, -0.06) | <0.001 | | -0.07 (-0.09, -0.05) | <0.001 | | |  |
| ^c^Vertical position - base, ED, mm | | 0.00 (-0.01, 0.01) | 0.96 | | 0.01 (0.00, 0.03) | 0.03 | | |  |
| ^d^Number of ROI knots, *N* | | 0.02 (-0.02, 0.07) | 0.33 | | 0.01 (-0.05, 0.06) | 0.82 | | |  |
| ^c^Syst. apical foreshortening, mm | | 0.41 (0.35, 0.48) | <0.001 | | 0.54 (0.47, 0.60) | <0.001 | | |  |
| **Apical long-axis view (N≥2,713 (96%))** | | | | | | | | | |
| ^a^ROI centerline length, ED, cm | | -0.03 (-0.09, 0.03) | 0,32 | | 0.09 (0.01, 0.17) | 0.03 | | |  |
| ^a^LV length by ROI, ED, cm | | -0.11 (-0.20, -0.02) | 0,02 | | 0.11 (-0.02, 0.25) | 0.09 | | |  |
| ^b^Averaged ROI width, ED, mm | | -0.67 (-0.75, -0.59) | <0.001 | | -0.59 (-0.68, -0.51) | <0.001 | | |  |
| ^c^Vertical position - apex, ED, mm | | -0.00 (-0.02, 0.01) | 0.88 | | 0.02 (0.00, 0.04) | 0.01 | | |  |
| ^c^Horizontal deviation - apex, ED, mm* | | -0.03 (-0.05, 0.01) | 0.01 | | -0.02 (-0.05, -0.01) | 0.01 | | |  |
| ^c^Vertical position - base, ED, mm | | -0.01 (-0.02, -0.00) | 0.01 | | -0.01 (-0.02, 0.01) | 0.29 | | |  |
| ^d^Number of ROI knots, *N* | | 0.12 (0.07, 0.17) | <0.001 | | 0.07 (0.02, 0.12) | 0.005 | | |  |
| ^c^Syst. apical foreshortening, mm | | 0.76 (0.68, 0.83) | <0.001 | | 0.87 (0.80, 0.94) | <0.001 | | |  |

*Supplementary Table S4. Analyses adjusted for: ^a^Age, sex and body surface area, ^b^age, systolic blood pressure and mean left ventricular wall thickness, ^c^body mass index, ^d^body mass index and image quality score. *Mean (SD), with negative values leftward for centerline, positive values rightward. Regression based on absolute values. Abbreviations: CI, confidence interval; ED, end-diastolic; LV, left ventricular; ROI, region of interest; SD, standard deviation.*

**Supplementary Table S5.** Distribution of between operator differences in in specific strain measurement procedure parameters.

| **Parameters (N≥1,333 (94%))** | **Average of views**  **Mean (SD)** | **A4C**  **Mean (SD)** | **A2C**  **Mean (SD)** | **ALAX**  **Mean (SD)** |
| --- | --- | --- | --- | --- |
| ^a^ROI centerline length, ED, cm | -0.50 (0.42) | -0.87 (0.56) | -0.60 (0.57) | -0.04 (0.69) |
| ^a^LV length by ROI, ED, cm | -0.16 (0.14) | -0.34 (0.23) | -0.17 (0.16) | 0.01 (0.26) |
| ^b^Averaged ROI width, ED, mm | 0.06 (0.76) | 0.05 (0.80) | -0.02 (0.84) | 0.13 (0.81) |
| ^c^Vertical position - apex, ED, mm | 0.22 (1.16) | 0.30 (1.53) | 0.38 (1.66) | -0.03 (1.61) |
| ^c^Horizontal deviation - apex, ED, mm* | 0.52 (1.66) | 0.02 (2.47) | 1.22 (2.77) | 0.33 (2.44) |
| ^c^Vertical position - base, ED, mm | -1.67 (1.38) | -3.48 (2.25) | -1.67 (1.60) | 0.13 (2.67) |
| ^d^Number of ROI knots, *N* | 0.02 (0.22) | 0.01 (0.30) | 0.02 (0.24) | 0.03 (0.31) |
| ^c^Syst. apical foreshortening, mm | 0.03 (0.47) | 0.03 (0.68) | 0.09 (0.77) | -0.01 (0.64) |

*Supplementary Table S5. GLS and analyses specific differences were processed as data from the expert minus data from the experienced or intermediate experienced operators. Positive β indicate higher GLS in % per unit difference in the specified parameters between the repeated analyses. Other explanations and abbreviations as in Supplementary tables S1 and S4.*

**Supplementary Table S6.** Associations of differences between operators in repeated analyses of left ventricular regional longitudinal strain from standard apical views with differences in specific strain measurement procedure parameters.

| **Parameters** | **Unadjusted  β (95% CI)** | ***P*_unadj._** | **Adjusted β (95% CI)** | ***P_adj._*** |  |
| --- | --- | --- | --- | --- | --- |
|  |  |  |  |  |  |
| **Apical 4-chamber view (N≥1,330 (93%))** | | | | | |
| ^a^ROI centerline length, ED, cm | -0.68(-0.85, -0.51) | <0.001 | -0.64 (-0.81, -0.46) | <0.001 |  |
| ^a^LV length by ROI, ED, cm | 0.00 (-0.43, 0.44) | 0.99 | 0.00 (-0.44, 0.43) | 0.98 |  |
| ^b^Averaged ROI width, ED, mm | -0.30 (-0.42, -0.18) | <0.001 | -0.31 (-0.43, -0.19) | <0.001 |  |
| ^c^Vertical position - apex, ED, mm | 0.35 (0.29, 0.41) | <0.001 | 0.35 (-0.06, 0.00) | 0.02 |  |
| ^c^Horizontal deviation - apex, ED, mm* | 0.03 (-0.01, 0.07) | 0.10 | 0.04 (0.00, 0.08) | 0.07 |  |
| ^c^Vertical position - base, ED, mm | -0.03 (-0.07, 0.01) | 0.17 | -0.04 (-0.08, 0.01) | 0.10 |  |
| ^d^Number of ROI knots, *N* | -0.17 (-0.49, 0.16) | 0.32 | -0.05 (-0.40, 0.29) | 0.76 |  |
| ^c^Syst. apical foreshortening, mm | 1.04 (0.91, 1.17) | <0.001 | 1.01 (0.88, 1.15) | <0.001 |  |
| **Apical 2-chamber view (N≥1,312 (93%))** | | | | | |
| ^a^ROI centerline length, ED, cm | | -0.84 (-1.02, -0.67) | <0.001 | -0.77 (-0.95, -0.60) | <0.001 |
| ^a^LV length by ROI, ED, cm | | -0.68 (-1.33, -0.03) | 0.04 | -0.56 (-1.21, 0.08) | 0.09 |
| ^b^Averaged ROI width, ED, mm | | -0.14 (-0.26, -0.02) | 0.02 | -0.16 (-0.28, -0.04) | 0.01 |
| ^c^Vertical position - apex, ED, mm | | 0.33 (0.27, 0.38) | <0.001 | 0.32 (0.26, 0.38) | <0.001 |
| ^c^Horizontal deviation - apex, ED, mm* | | 0.12 (0.08, 0.15) | <0.001 | 0.11 (0.08, 0.15) | <0.001 |
| ^c^Vertical position - base, ED, mm | | -0.12 (-0.19, -0.06) | <0.001 | -0.13 (-0.20, -0.07) | <0.001 |
| ^d^Number of ROI knots, *N* | | -0.06 (-0.50, 0.38) | 0.80 | -0.01 (-0.48, 0.47) | 0.98 |
| ^c^Syst. apical foreshortening, mm | | 0.80 (0.67, 0.92) | <0.001 | 0.77 (0.65, 0.90) | <0.001 |
| **Apical long-axis view (N≥1,307 (93%))** | | | | | |
| ^a^ROI centerline length, ED, cm | -0.55 (-0.69, -0.41) | <0.001 | -0.49 (-0.63, -0.35) | <0.001 |  |
| ^a^LV length by ROI, ED, cm | 0.11 (-0.26, 0.49) | 0.55 | 0.22 (-0.15, 0.58) | 0.25 |  |
| ^b^Averaged ROI width, ED, mm | -0.08 (-0.20, 0.04) | 0.19 | -0.09 (-0.21, 0.03) | 0.13 |  |
| ^c^Vertical position - apex, ED, mm | 0.43 (0.38, 0.49) | <0.001 | 0.43 (0.37, 0.49) | <0.001 |  |
| ^c^Horizontal deviation - apex, ED, mm* | 0.05 (0.01, 0.09) | 0.01 | 0.05 (0.01, 0.09) | 0.01 |  |
| ^c^Vertical position - base, ED, mm | -0.03 (-0.06, 0.01) | 0.15 | -0.02 (-0.06, 0.01) | 0.18 |  |
| ^d^Number of ROI knots, *N* | -0.04 (-0.35, 0.27) | 0.79 | -0.01 (-0.33, 0.30) | 0.93 |  |
| ^c^Syst. apical foreshortening, mm | 1.04 (0.90, 1.19) | <0.001 | 1.00 (0.86, 1.14) | <0.001 |  |

*Supplementary Table S6. Explanations and abbreviations as in Supplementary tables S2 and S5.*
